# Effective gene editing by high-fidelity base editor 2 in mouse zygotes

**DOI:** 10.1007/s13238-017-0418-2

**Published:** 2017-06-05

**Authors:** Puping Liang, Hongwei Sun, Ying Sun, Xiya Zhang, Xiaowei Xie, Jinran Zhang, Zhen Zhang, Yuxi Chen, Chenhui Ding, Yuanyan Xiong, Wenbin Ma, Dan Liu, Junjiu Huang, Zhou Songyang

**Affiliations:** 1 Key Laboratory of Gene Engineering of the Ministry of Education, Guangzhou Key Laboratory of Healthy Aging Research and State Key Laboratory of Biocontrol, SYSU-BCM Joint Research Center, School of Life Sciences, Sun Yat-sen University, Guangzhou, 510275 China; 2 State Key Laboratory of Ophthalmology, Zhongshan Ophthalmic Center, Sun Yat-sen University, Guangzhou, 510275 China; 3 Key Laboratory of Reproductive Medicine of Guangdong Province, School of Life Sciences and the First Affiliated Hospital, Sun Yat-sen University, Guangzhou, 510275 China; 4 Guangzhou Magigen Biotechnology Co.Ltd, Guangzhou, 510320 China; 5 Verna and Marrs Mclean Department of Biochemistry and Molecular Biology, Baylor College of Medicine, One Baylor Plaza, Houston, TX 77030 USA

**Keywords:** base editor, high-fidelity, mouse embryos, proximal-site deamination, whole-genome sequencing

## Abstract

Targeted point mutagenesis through homologous recombination has been widely used in genetic studies and holds considerable promise for repairing disease-causing mutations in patients. However, problems such as mosaicism and low mutagenesis efficiency continue to pose challenges to clinical application of such approaches. Recently, a base editor (BE) system built on cytidine (C) deaminase and CRISPR/Cas9 technology was developed as an alternative method for targeted point mutagenesis in plant, yeast, and human cells. Base editors convert C in the deamination window to thymidine (T) efficiently, however, it remains unclear whether targeted base editing in mouse embryos is feasible. In this report, we generated a modified high-fidelity version of base editor 2 (HF2-BE2), and investigated its base editing efficacy in mouse embryos. We found that HF2-BE2 could convert C to T efficiently, with up to 100% biallelic mutation efficiency in mouse embryos. Unlike BE3, HF2-BE2 could convert C to T on both the target and non-target strand, expanding the editing scope of base editors. Surprisingly, we found HF2-BE2 could also deaminate C that was proximal to the gRNA-binding region. Taken together, our work demonstrates the feasibility of generating point mutations in mouse by base editing, and underscores the need to carefully optimize base editing systems in order to eliminate proximal-site deamination.

**Electronic supplementary material:**

The online version of this article (doi:10.1007/s13238-017-0418-2) contains supplementary material, which is available to authorized users.

## INTRODUCTION

The human genome project has revealed unprecedented genetic diversity in human, manifested predominantly as single nucleotide variations (SNVs). Probing the physiological significance of these SNVs is both essential and challenging. Researchers have traditionally relied on homologous recombination (HR) to generate SNVs, a process that is inefficient (usually <10^−5^), labor-intensive, and often ineffective in non-dividing primary cells (Capecchi, 2005; Porteus and Carroll, 2005; Thomas and Capecchi, 1987). Molecular scissors such as ZFN, TALEN, and CRISPR/Cas9 can promote HR at target sites, however, the more efficient non-homologous end joining (NHEJ) pathway invariably outcompetes HR in these cases (Cho et al., 2013; Cong et al., 2013; Gaj et al., 2013; Jiang et al., 2013; Jinek et al., 2013; Kim and Kim, 2014; Komor et al., 2017; Porteus, 2006; Suzuki et al., 2016; Tesson et al., 2011; Yang et al., 2013). A programmable cytidine deaminase built on the CRISPR/Cas9 platform has recently been developed to more efficiently edit target bases (Komor et al., 2016; Ma et al., 2016; Nishida et al., 2016). This base editor (BE) has an effector that fuses cytidine deaminase (rAPOBEC1) with Cas9 and the uracil DNA glycosylase inhibitor (UGI), enabling targeted cytidine (C) to uridine (U) conversion in the desired DNA sequence (Komor et al., 2016; Ma et al., 2016; Nishida et al., 2016). Following DNA replication, this conversion will lead to C-to-T (or G-to-A) substitution. BE-directed base editing at single-base resolution has been successfully carried out in plant, yeast, and human cells (Komor et al., 2016; Li et al., 2016; Lu and Zhu, 2016; Nishida et al., 2016), and shown to be >100-fold more efficient than HR at generating point mutations (Komor et al., 2016; Ma et al., 2016; Nishida et al., 2016). It has also been found that base editors could efficiently deaminate cytidines within a deamination window, typically several nucleotides long (positions 4–8) in the gRNA-binding region (Komor et al., 2016). Previous studies using CRISPR-based genome editing methods to generate point mutations in mice could not achieve 100% efficiency and resulted in mosaicism (Inui et al., 2014; Wu et al., 2013). Whether BE-mediated genome editing proves more efficient and reliable remains to be studied.

Of the different base editors, base editor 3 (BE3, rAPOBEC1-nCas9-UGI) uses the Cas9 nickase (nCas9, D10A), whereas base editor 2 (BE2, rAPOBEC1-dCas9-UGI) utilizes the nuclease activity dead Cas9 mutant (dCas9, D10A/H840A). In cells, BE2 appeared to have lower base editing efficiency than BE3, although still more efficient than HR. Unlike nCas9, dCas9 does not cleave DNA, which should help reduce off-target indels and increase the specificity of BE2. Since the gRNA/Cas9 units within base editors are responsible for their targeting, improving Cas9 specificity, such as using high-fidelity Cas9 variants, should improve the specificity of base editors and further reduce off-targets. The Cas9 high-fidelity 1 variant (Cas9-HF1), which contains four point mutations (N497A/R661A/Q695A/Q926A), is thought to have less binding energy with DNA than wild type Cas9. The mutations presumably disrupt hydrogen bonding with the phosphate backbone of the complementary DNA strand, thereby decreasing Cas9 binding with mismatched sequences and increasing its overall specificity (Anders et al., 2014; Kleinstiver et al., 2016; Nishimasu et al., 2014). Cas9 high-fidelity 2 (Cas9-HF2), which contains one additional mutation (D1135E) compared to Cas9-HF1 and exhibits altered PAM preference (from NGG/A to NGG only), has been proven highly specific based on genome-wide sequencing and targeted deep sequencing analyses (Kleinstiver et al., 2016; Kleinstiver et al., 2015). We have generated a high-fidelity variant of base editor 2 (HF2-BE2) by introducing the five point mutations into dCas9 (Kleinstiver et al., 2016). Here, we report our findings on using HF2-BE2 to edit target genes in mouse zygotes.

We found that both HF2-BE2 and BE2 could convert target C to T efficiently in mouse embryos, where the editing efficiency of HF2-BE2 appeared higher than that of BE2. We found biallelic mutant embryos and pups, indicating 100% efficiency in base conversion. Moreover, we found that both HF2-BE2 and BE2 could deaminate cytidines on non-target strand as well as target strand. Surprisingly, both HF2-BE2 and BE2 could deaminate C proximal to the deamination window, which we termed proximal-site deamination. Taken together, our data highlight the potential of base editors in generating point mutations in mouse, and underscore the need to optimize base editors in order to avoid proximal-site deamination.

## RESULTS

### The high-fidelity version of BE2 (HF2-BE2) mediates efficient editing in mouse embryos

**Figure 1:**
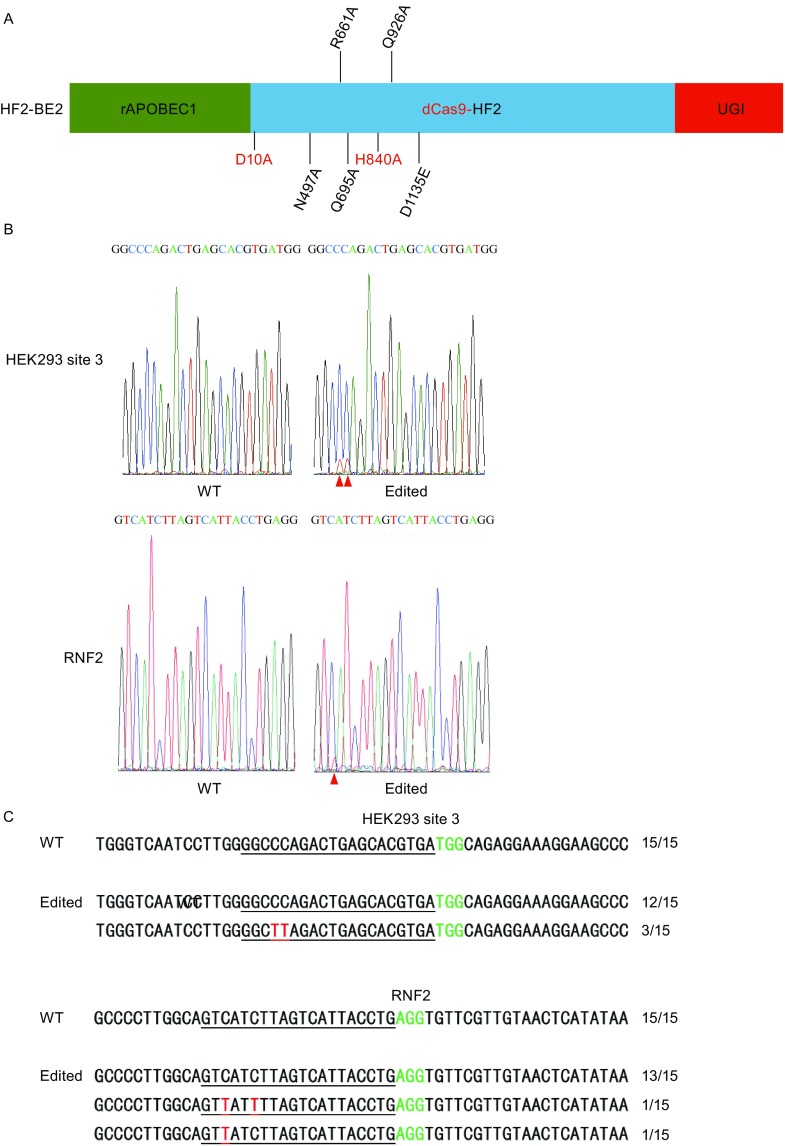
**HF2-BE2 edits target bases efficiently in human cells**. (A) Schematic representation of HF2-BE2. Point mutations that inactivate Cas9 nuclease activity are shown in red, and mutations that enhance its fidelity are shown in black. (B) The HF2-BE2 expression vector was respectively co-transfected into 293T cells with gRNA expression vectors targeting HEK293 site 3 and RNF2 (Komor et al., 2016). Genomic DNA was extracted from the edited cell populations for PCR amplification of the target sites. Sequencing chromatographs of the PCR amplicons are shown. WT, untransfected wild-type control cells. Edited, base-edited cells. Red arrowheads, successfully edited base. (C) The PCR amplicons from (B) were subcloned into pGEM-T vectors and sequenced. The number of clones for each sequence pattern is indicated. Underlined, gRNA target regions. Green, PAM sequence. Red, point mutations

We first examined the ability of HF2-BE2 (rAPOBEC1-XTEN-dCas9-HF2-UGI) (Fig. 1A) to edit two previously published base editor target sites in human cells (HEK293 site 3 and RNF2) (Komor et al., 2016), by co-transfecting HF2-BE2 with the respective gRNAs into 293T cells. Genomic DNA analysis clearly indicated the presence of thymidine peaks in the target region (Fig. 1B). PCR amplicons of the target sequences from both untransfected wild-type (WT) control cells and edited cells were then subcloned, and 15 single bacterial clones from each pool were sequenced (Fig. 1C). As expected, both wildtype and deaminated alleles were found in the edited cells, indicating successful base editing by HF2-BE2.

**Figure 2:**
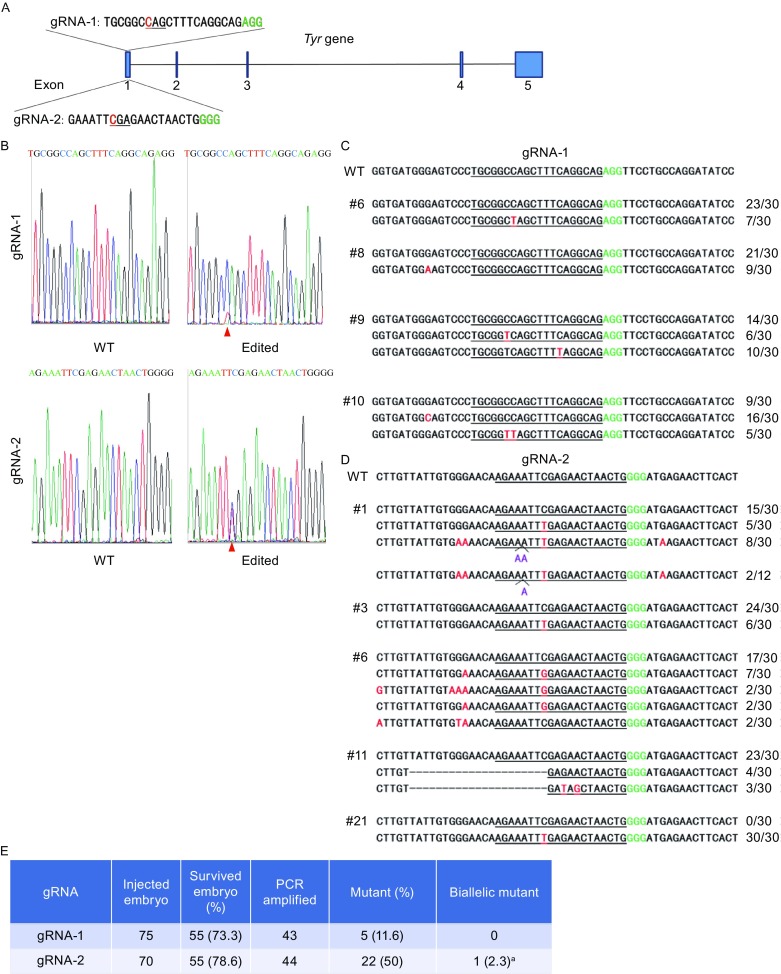
**HF2-BE2 mediates efficient base editing of** ***Tyr*** **in mouse embryos**. (A) Schematic representation of the *Tyr* locus and gRNA target sites. gRNA target sequences are also shown. The codon to be modified is underlined, with the nucleotide to be deaminated in red. The protospacer adjacent motif (PAM) is in green. (B) The two gRNAs were respectively co-injected into 1-cell zygotes with HF2-BE2 mRNA, and the embryos were analyzed 48 h later. Representative sequencing chromatographs of the PCR amplicons of target sites are shown here. WT, wild-type embryo. Edited, embryos edited by HF2-BE2 with the successfully edited base indicated by red arrowheads. (C) PCR amplicons of gRNA-1 target site from the genomic DNA of selected embryos were subcloned into pGEM-T vectors and sequenced. The number of clones for each sequence pattern is indicated. Underlined, gRNA target regions. Green, PAM sequence. Red, point mutations. Purple, insertions. Dash, deletions. (D) PCR amplicons of gRNA-2 target site from the genomic DNA of selected embryos were subcloned into pGEM-T vectors and sequenced. (E) Summary of base editing by HF2-BE2 in mouse embryos. a, this biallelic mutant embryo is homozygous

We next investigated HF2-BE2-mediated base editing in mouse embryos, by generating two gRNAs targeting exon 1 of the *Tyr* gene (Fig. 2A) and respectively co-injecting them into the cytoplasm of 1-cell zygotes with HF2-BE2 mRNA. The injected embryos were harvested after 48 h for genomic DNA extraction and genotyping by Sanger sequencing (Fig. 2B) and subcloning/sequencing analysis (Fig. 2C and 2D). Both gRNAs were able to direct efficient C–T conversion on the non-target strand in the target region (Fig. 2C and 2D), and to a lesser extent, C–G/A conversion (Figs. 2C, 2D, and S1). Unexpectedly, we found C–T conversion on the target strand and deamination at cytidines proximal to gRNA binding sites (which we termed proximal-site deamination), even at cytidines 38 bps upstream or 3 bps downstream of the gRNA target site (Figs. 2C, 2D, and S1). In addition, we found indels in two embryos edited by gRNA-2 (#1 and #11) (Fig. 2D). Given the absence of nuclease activity in dCas9, these findings suggest that cytidine deamination alone can result in indels. Of the examined embryos, 11.6% and 50% respectively were edited by gRNA-1 and gRNA-2 (Fig. 2E). One gRNA-2 edited embryo was a homozygous mutant (#21), indicating 100% base editing efficiency (Figs. 2D, 2E, and S2). Genomic DNA from this homozygous mutant embryo was further examined by whole-genome sequencing, which found no off-target deamination, suggesting that HF2-BE2 was able to bind specifically the target site in mouse embryos (Table S1).

### One-step generation of base-edited mouse by HF2-BE2

**Figure 3:**
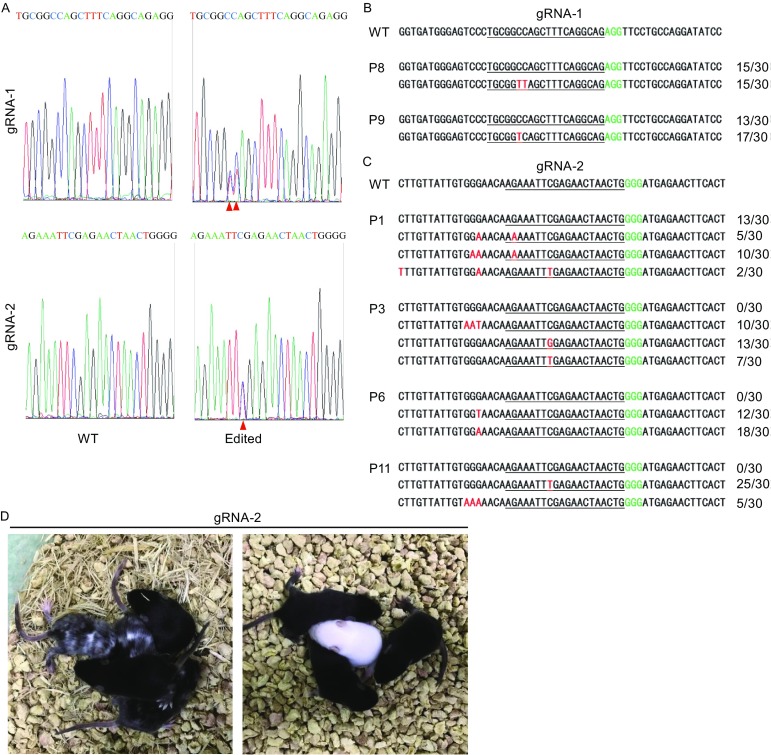
**Generation of base-edited mice using HF2-BE2**. (A) Representative sequencing chromatographs of the PCR amplicons of target sites from the founder mice are shown here. WT, wild-type embryo. Edited, embryo edited by HF2-BE2, with the successfully edited base indicated by red arrowheads. (B) PCR amplicons of gRNA-1 target sites from the genomic DNA of selected founder mice were subcloned into pGEM-T vectors and sequenced. The number of clones for each sequence pattern is indicated. Underlined, gRNA target regions. Green, PAM sequence. Red, point mutations. (C) PCR amplicons of gRNA-2 target sites from the genomic DNA of selected founder mice were subcloned into pGEM-T vectors and sequenced. (D) Founder pups (10 days old) from the gRNA-2 group

We then proceeded to generate base-edited mice. To rule out possible embryonic toxicity and better determine base editing efficacy, HF2-BE2 mRNA was injected alone or together with gRNA-1 or gRNA-2. Nuclease-free water was also included as a control. The injected embryos were then transplanted into pseudopregnant mice. The rate of pups obtained after transplantation appeared similar between different groups (Table 1), indicating low toxicity of HF2-BE2. Genotyping revealed that 2 out of 11 (18.2%) pups from gRNA-1 group and 7 out of 11 (63.6%) pups from gRNA-2 group were mutants (Table 1, Figs. 3A–C, S3, and S4). Of the gRNA-2 group, we obtained 3 (27.3%) biallelic mutant founder mice (P3, P6, P11), in line with the mouse embryo data (Fig. 2E and Table 1). Furthermore, as was observed in mouse embryos (Fig. 2C and 2D), C-to-T conversion occurred on both target and non-target strands in founder mice (Fig. 3C). Additionally, we also found proximal-site deamination in 4 pups (Fig. 3C), one of which lies 42 bps downstream of the PAM sequence on the target strand (Fig. S5).

**Table 1:** Summary of base editing by HF2-BE2 in founder mice

Group	Survived/Injected embryos (%)	Pups/Transferred (%)	Albino pups (%)	Mosaic pups (%)	Mutant black pups (%)	Mutants (%)	Biallelic mutants (%)
gRNA-1 + HF2-BE2 mRNA^*^	120/162 (74.1)	13/120 (10.8)	0	0	2 (18.2) ^*^	2 (18.2)	0
gRNA-2 + HF2-BE2 mRNA	106/145 (73.1)	11/106 (10.4)	1 (9.1)	4 (36.4)	2 (18.2)	7 (63.6)	3 (27.3)
HF2-BE2 mRNA^#^	108/142 (76.1)	14/108 (13.0)	0	0	0	0	0
H_2_O	103/146 (70.5)	9/103 (8.7)	0	0	0	0	0

^*, #^Pups were cannibalized by the mother (2 for * and 1 for ^#^)

Successful C-to-T conversion by HF2-BE2 is expected to yield a premature stop codon in the gRNA target regions, leading to albinism in C57B/6 J mice. Two black pups from gRNA-1 group (P8 and P9) showed ~50% base editing efficiency, suggesting that they were heterozygous mutants. Of the 11 pups in gRNA-2 group, 4 (P1, P3, P5, P11) were chimeras (coat-color mosaic) and 1 (P6) was albino (Fig. 3D). In this albino pup, the GAA codon (Gln) −2 bp from the gRNA target site was converted respectively to TAA (stop) and AAA (lysine), implicating this glutamine residue as being critical for the activity and function of tyrosinase (Fig. 3C). When the biallelic mutant P11 pup was mated with WT mouse, we found that the mutant allele could be successfully transmitted to the next generation (Fig. S6).

### Proximal-site deamination of cytidines near gRNA binding sites by HF2-BE2

**Figure 4:**
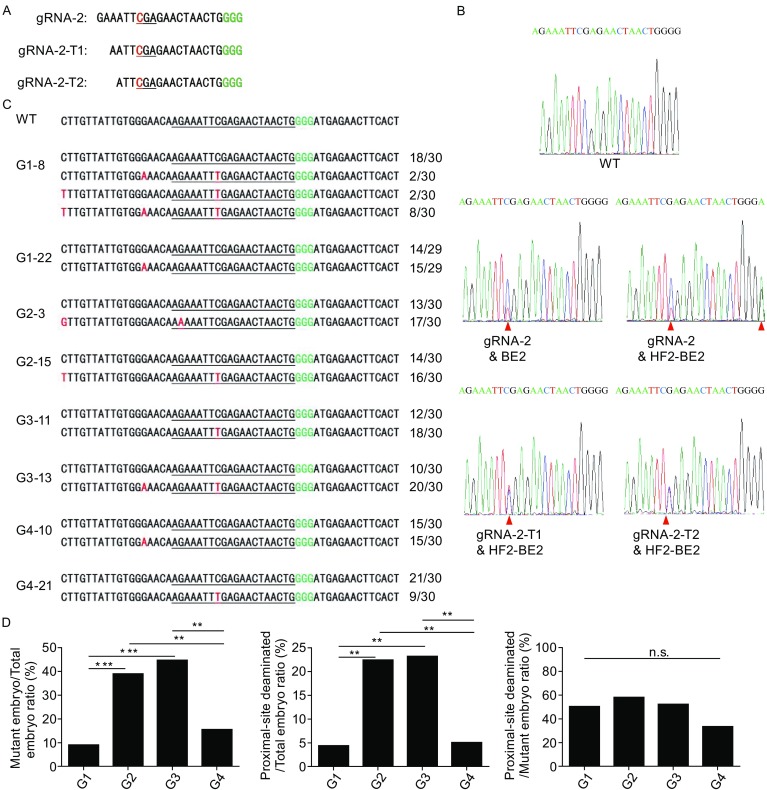
**Examination of proximal-site deamination by HF2-BE2**. (A) Design of truncated gRNA variants of gRNA-2 (gRNA-2-T1 and gRNA-2-T2). The codon to be modified is underlined, with the nucleotide to be deaminated in red. PAM is in green. (B) Representative sequencing chromatographs of the PCR amplicons of target sites from the embryos are shown here. Bases successfully edited are indicated by red arrowheads. (C) PCR amplicons of target sites from embryos were subcloned into pGEM-T vectors and sequenced. The number of clones for each sequence pattern is indicated. Underlined, gRNA target regions. Green, PAM sequence. Red, point mutations. (D) Statistical analysis of base editing by HF2-BE2 and BE2 in mouse embryos. n.s., not significant, *P* values > 0.05. ^**^ *P* < 0.01, ^***^ *P* < 0.001, statistical significance was determined using the χ^2^ test

It is postulated that rAPOBEC1 catalyzes C-to-T conversions at exposed single-stranded DNA regions displaced by gRNAs (Conticello, 2008; Harris et al., 2002; Komor et al., 2016; Saraconi et al., 2014), truncating gRNAs may therefore reduce proximal-site deamination by unwinding smaller stretches of DNA (Fu et al., 2014a; Fu et al., 2014b). To better assess how manipulating gRNA/dCas9 targeting affects the activity of HF2-BE2, we generated two truncated versions of gRNA-2 (gRNA-2-T1 and gRNA-2-T2) with 16 or 17 nucleotides of guide sequence (Fig. 4A). HF2-BE2 mRNA was individually co-injected into mouse embryos with gRNA-2, gRNA-2-T1, and gRNA-2-T2 (groups 2, 3, & 4, G2, 3, & 4). In addition, we also co-injected conventional BE2 mRNA (containing no HF mutations) with gRNA-2 (group 1, G1) as a comparison. The embryos were genotyped 48 h after injection (Fig. 4B and 4C). Of the different combinations, group 2 with HF2-BE2 mRNA and full-length gRNA-2 clearly had higher editing efficiency, as well as the highest number of proximal-site base edits (Fig. 4D and Table 2). However, the ratio of proximal-site deaminated embryos *vs.* mutant embryos was similar between groups 1 and 2, suggesting that lower proximal-site deamination in BE2-edited embryos was most likely a result of lower overall deamination activity of BE2 compared to HF2-BE2. While group 3 showed similar editing efficiency as group 2, group 4 was significantly reduced, suggesting that excessive truncation of gRNA sequences drastically decreased the efficiency of base editing by HF2-BE2 (Fig. 4D). Moreover, the differences in the ratios of proximal-site deaminated/mutant embryo were not statistically significant between the groups (*P* values > 0.05), implying that truncating gRNAs will decrease the efficiency of on-target as well as proximal-site deamination, consistent with findings in human cells (Kim et al., 2017b). These data demonstrate that base editors may deaminate cytidines proximal to gRNA binding sites, similar to observations in *E*. *coli* cells, where the zinc-finger domain-guided cytidine deaminase induced off-target deamination at cytidines ~150-bp away from the target site (Yang et al., 2016).

**Table 2:** Summary of base editing by HF2-BE2 and BE2 in mouse embryos

Group	gRNA + base editor	Total embryos	Mutant embryos (%)	Proximal-site deaminated embryos	Proximal-site deaminated/ Mutant embryos ratio (%)	Proximal-site deaminated/ Total embryos ratio (%)
1	gRNA-2 + BE2 mRNA	68	6 (8.8)	3	50.0	4.4
2	gRNA-2 + HF2-BE2 mRNA	67	26 (38.8)	15	57.7	22.4
3	gRNA-2-T1 + HF2-BE2 mRNA	56	25 (44.6)	13	52.0	23.2
4	gRNA-2-T2 + HF2-BE2 mRNA	59	9 (15.3)	3	33.30	5.1

## DISCUSSION

In this study, we present data that highlight important similarities and differences between base editors. While this manuscript was under preparation, Kim et al. reported generating fully base-edited mouse embryos using BE3 (Kim et al., 2017a). Similar to base editor 3 (BE3), both BE2 and HF2-BE2 could efficiently modify bases in mouse zygotes. Indeed, we were able to obtain homozygous edited embryos free of mosacism using HF2-BE2 (Fig. 2D). Unlike BE3, BE2 and HF2-BE2 are able to convert C to T on both strands. This difference is likely caused by the ability of BE3 to cleave the modified target strand, rendering it incapable of serving as a DNA repair template. Base editing by deaminases is constrained by the presence of cytidines on a given target. Our data suggest that BE2 and HF2-BE2 may expand the choice of target nucleotides thanks to their abilities to deaminate C on both strands of target DNA. Previous studies using BE3 did not find proximal-site deamination (Kim et al., 2017a; Komor et al., 2016; Li et al., 2016; Lu and Zhu, 2016; Zong et al., 2017). In this study, we found that BE2 and HF2-BE2 could both lead to proximal-site deamination at cytidines close to gRNA target sites, which may occur as a result of spontaneous or Cas9-catalyzed DNA unwinding that exposes single-stranded DNA to rAPOBEC1. To avoid proximal-site deamination, reducing the amount of HF2-BE2 (or BE2) mRNAs and gRNA and/or truncating gRNAs may be useful at some sites (Kim et al., 2017b). Recently, a base editor variant with a narrower deamination window was described, such modifications may bring more specificity without sacrificing efficiency at genomic sites (Kim et al., 2017b). Additionally, we found higher editing efficiency for HF2-BE2 than BE2 in mouse embryos, when guided by full-length gRNA-2. A more comprehensive comparison of HF2-BE2 *vs.* BE2 and other base editors is needed to fully understand the effects of different Cas9 proteins on the efficiency and specificity of base editors.

We were surprised to find low-frequency (2/44 embryos) base insertions and deletions at target sites with HF2-BE2, even though dCas9 has no demonstrable nuclease activity (Hsu et al., 2014; Mali et al., 2013; Qi et al., 2013). Such indels are unlikely the result of any potential residual enzymatic activities of dCas9, because they were far from Cas9 cleavage sites (≥12 bp upstream of the PAM sequence) (Jinek et al., 2012). Taken together with previous findings that fusing UGI to nCas9-PmCDA1 suppressed indel formation (Nishida et al., 2016), we hypothesize that the indels might have been caused by deamination and base excision repair. If the base excision repair pathway is active when deamination occurs simultaneously on both strands, base excision will create abasic sites on both strands and ultimately DNA double-strand breaks (DSB) (Kingma et al., 1995; Ma et al., 2009). It has been shown that even a single abasic site is capable of inducing DNA DSBs (Kidane et al., 2014). Subsequent DSB repair can result in indel formation. Consequently, to elucidate how DNA repair machineries are utilized following base conversions will be crucial to reducing and eliminating unwanted indels in genome editing mediated by base editors. Our study highlights the advantages of gene editing using base editor 2 and sheds light on possible new avenues of research for precise gene editing.

## MATERIALS AND METHODS

### Plasmids

BE2 and HF2-BE2 expression cassette were synthesized and ligated into pcDNA3.1 (-) vector by IGE BIOTECHNOLOGY LTD. To construct gRNA expression vector, gRNA backbone with U6 promoter were amplified from pX330, and then ligated into the pGEM-T vector (Promega).

### Animals

All animal experiments were performed according to protocols approved by the Committee on Animal Care at the School of Life Sciences, Sun Yat-Sen University. Superovulated C57BL/6J mice (6–8 week old females) were mated with C57BL/6 J males. Plugged females were sacrificed by cervical dislocation. Zygotes (0.5 day) were collected using potassium simplex optimized medium (KSOM) containing N′-2-Hydroxyethylpiperazine-N′-2-ethanesulfonic acid and sodium bicarbonate (HKSOM), and cultured in KSOM until genotyping or transplantation. Embryos were *in vitro* cultured for 48 h before genotyping or whole genome amplification. CD1 female mice (6–8 weeks old) that were mated with sterilized CD1 male mice were used as foster mothers.

### *In vitro* transcription

HF2-BE2 mRNA was transcribed using the mMESSAGE mMACHINE T7 ULTRA kit (Life Technologies) following the manufacturer’s instruction. gRNA-1 and gRNA-2 (Table S2) were cloned into the pDR274 vector (Addgene) and transcribed using the MEGAshortscript T7 kit (Life Technologies) following the manufacturer’s instruction. mRNAs and gRNAs were subsequently purifed using the MEGAclear kit (Life Technologies) and resuspended in RNase-free water.

### Intracytoplasmic injection of HF2-BE2 mRNA and gRNA

The mixture of HF2-BE2 mRNA (200 ng/μL) and gRNA (100 ng/μL) was injected into 0.5-day 1-cell zygotes of C57BL/6 J mice. The injected zygotes were transplanted into the oviduct of 0.5-day pseudopregnant mothers ~2 h after injection.

### Single embryo PCR amplification and mouse genotyping

Single embryo PCR amplification was performed as described before (Zhang et al., 2016). Briefly, each embryo was transferred into a PCR tube containing 1 μL lysis buffer, and then incubated at 65°C for 3 h followed by 95°C for 10 min. The lysis product was then amplified using primers listed in Table S3. Mouse genotyping was done by PCR and sequencing of tail-snips using the Mouse Genotyping Kit (KAPA Biosystems) and primers listed in Table S2.

### Genomic DNA analysis

Target sites were PCR amplified using primers listed in Table S2. The PCR products were then used in T7 endonuclease I (T7EI) cleavage assay as described before (Zhang et al., 2016). Primers for direct sequencing of the PCR products, which reveal the presence of double peaks and/or indels, are listed in Table S3. PCR products with double peaks were then TA cloned into the pGEM-T vector (Promega) for plasmid DNA extraction and Sanger sequencing.

### Whole genome sequencing, data processing, and off-target analysis

Whole genome amplification of embryos was performed using the PEPLI-g Midi Kit (Qiagen). Briefly, embryos were transferred into PCR tubes containing reconstituted buffer D2 (7 μL), and then incubated at 65°C for 10 min, before the addition of stop solution (3.5 μL) and MDA master mix (40 μL) and incubation at 30°C for 16 h. Whole genome sequencing (WGS) was done on an Illumina HiSeq 2000 PE150 as paired-end 150 bp reads. The reads were aligned to the mouse reference genome (UCSC, mm10) by means of BWA with default parameters (v0.7.13) (Li and Durbin, 2010). Samtools (v1.3, http://samtools.sourceforge.net) and Picard tools (version 2.2.2, http://picard.sourceforge.net) were used to build indices and remove duplicates. Base score recalibration (BaseRecalibrator) was applied by GATK (The Genome Analysis ToolKit, version 3.5-0) (McKenna et al., 2010) to enhance accuracy in identifying indels and single nucleotide variants (SNVs). GATK HaplotypeCaller was used to call variants for two samples and the variants were then divided into indels and SNVs by SelectVariants. Low-quality variants (indels and SNVs) and those appeared in dbSNP (build 142) were marked by VariantFiltration and discarded by Python.

To avoid false positive calls that overlap with repeat sequences and/or include homopolymers (Bansal and Libiger, 2011), we removed indels and SNVs that overlapped with low-complexity regions as defined by RepeatMasker (UCSC Genome Browser) and filtered out indels and SNVs containing homopolymers (>7 bp) in the low-complexity flanking region (±100 bp). To more definitively assign indels and SNVs, we searched regions flanking potential indel or SNV sites (±100 bp) for possible off-target sites. Bowtie1 (version 1.1.2, http://bowtie-bio.sourceforge.net) was used to align gRNA sequences (20 bp) to the ±100 bp sequences, allowing for ≤5 mismatches or perfect match of the last 10 nt 3′ of the gRNA. Successfully aligned sites with an NGG PAM were deemed as on/off-target sites. No potential off-target site indel or SNV was found in the homozygous embryo (Table S1).

## Electronic supplementary material

Below is the link to the electronic supplementary material.

Supplementary material 1 (PPTX 2975 kb)

Supplementary material 2 (XLSX 11 kb)

Supplementary material 3 (XLSX 10 kb)

Supplementary material 4 (XLSX 9 kb)
