# Supplementary material for: Effective gene editing by high-fidelity base editor 2 in mouse zygotes
Source: Protein Cell. 2017 Jun 5;8(8):601–11. doi: 10.1007/s13238-017-0418-2 (PMC5546933; doi:10.1007/s13238-017-0418-2)
Supplement: Supplementary file 1 — Supplementary material 1 (PPTX 2975 kb) [file 13238_2017_418_MOESM1_ESM.pptx]

## Slide 1
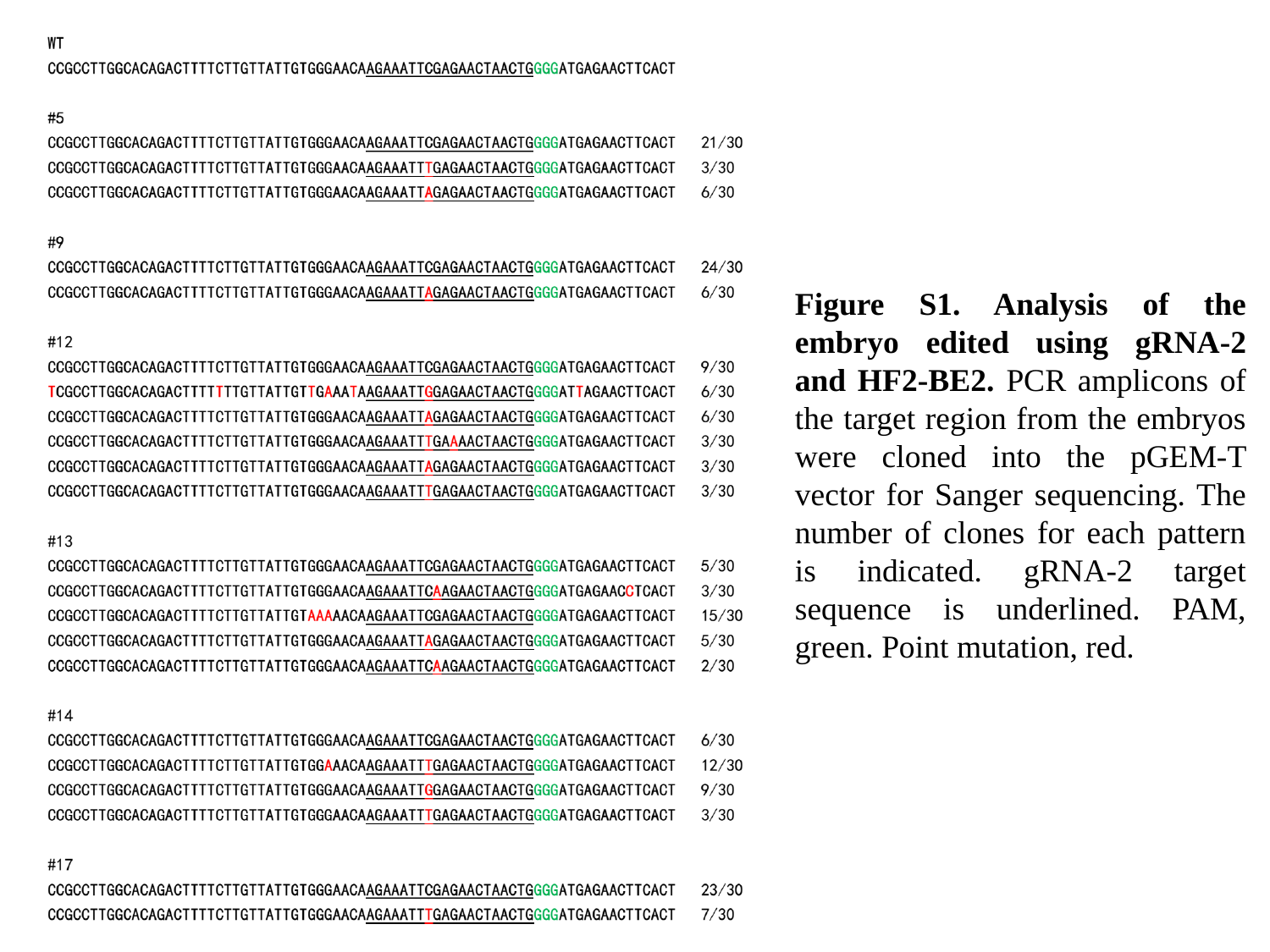

Figure S1. Analysis of the embryo edited using gRNA-2 and HF2-BE2. PCR amplicons of the target region from the embryos were cloned into the pGEM-T vector for Sanger sequencing. The number of clones for each pattern is indicated. gRNA-2 target sequence is underlined. PAM, green. Point mutation, red.

## Slide 2
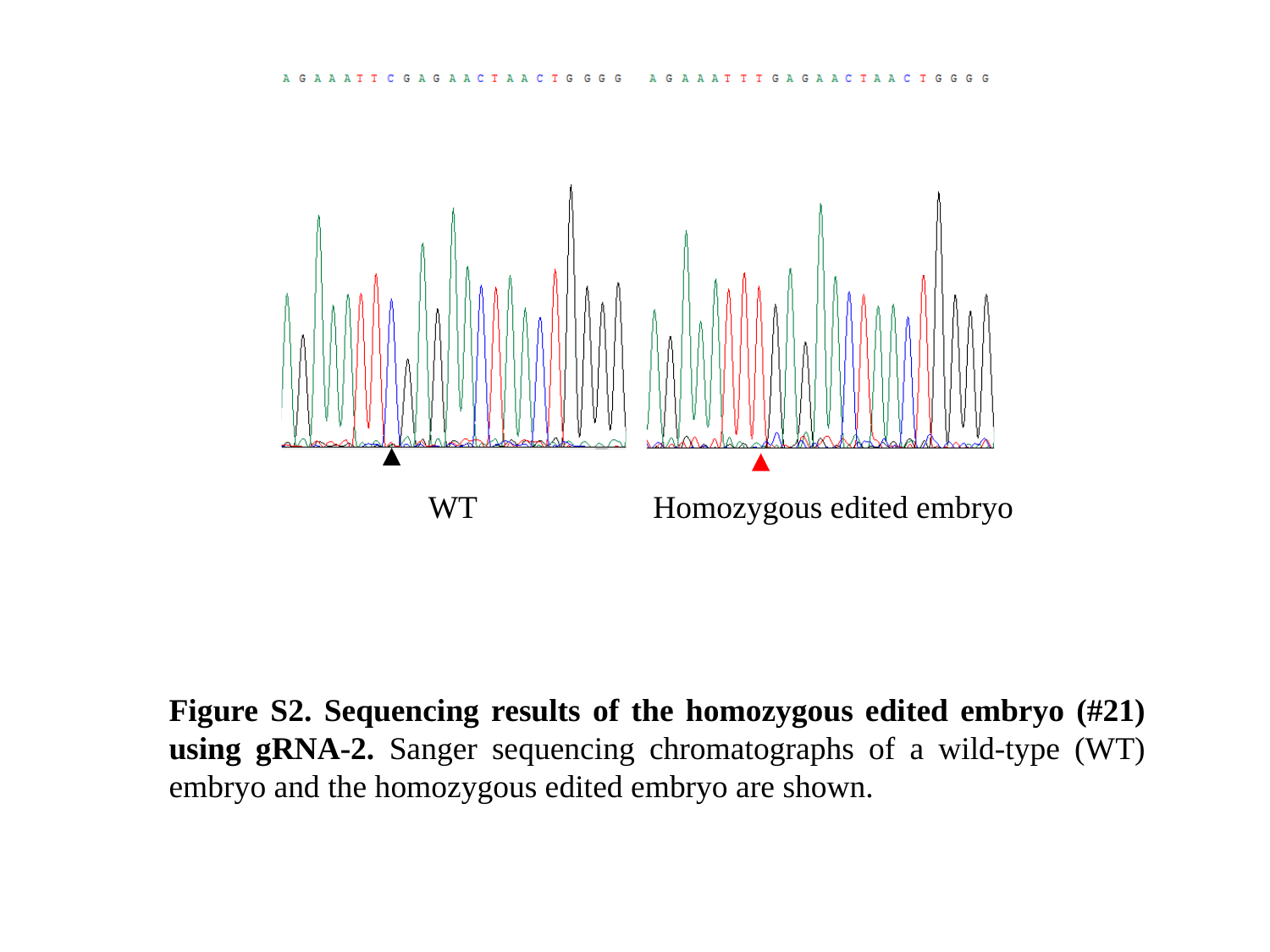

WT
Homozygous edited embryo
Figure S2. Sequencing results of the homozygous edited embryo (#21) using gRNA-2. Sanger sequencing chromatographs of a wild-type (WT) embryo and the homozygous edited embryo are shown.

## Slide 3
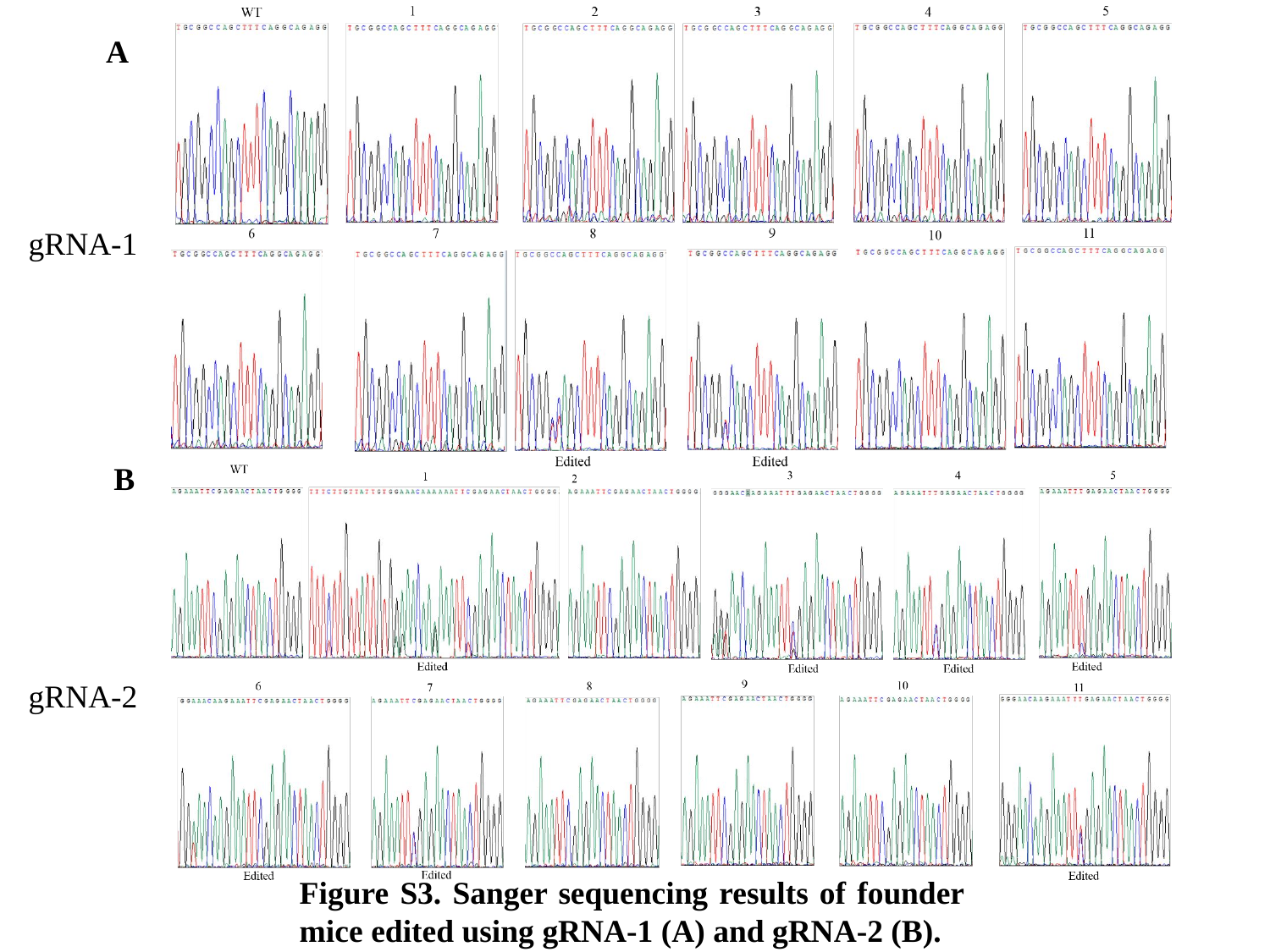

A
gRNA-1
B
gRNA-2
Figure S3. Sanger sequencing results of founder mice edited using gRNA-1 (A) and gRNA-2 (B).

## Slide 4
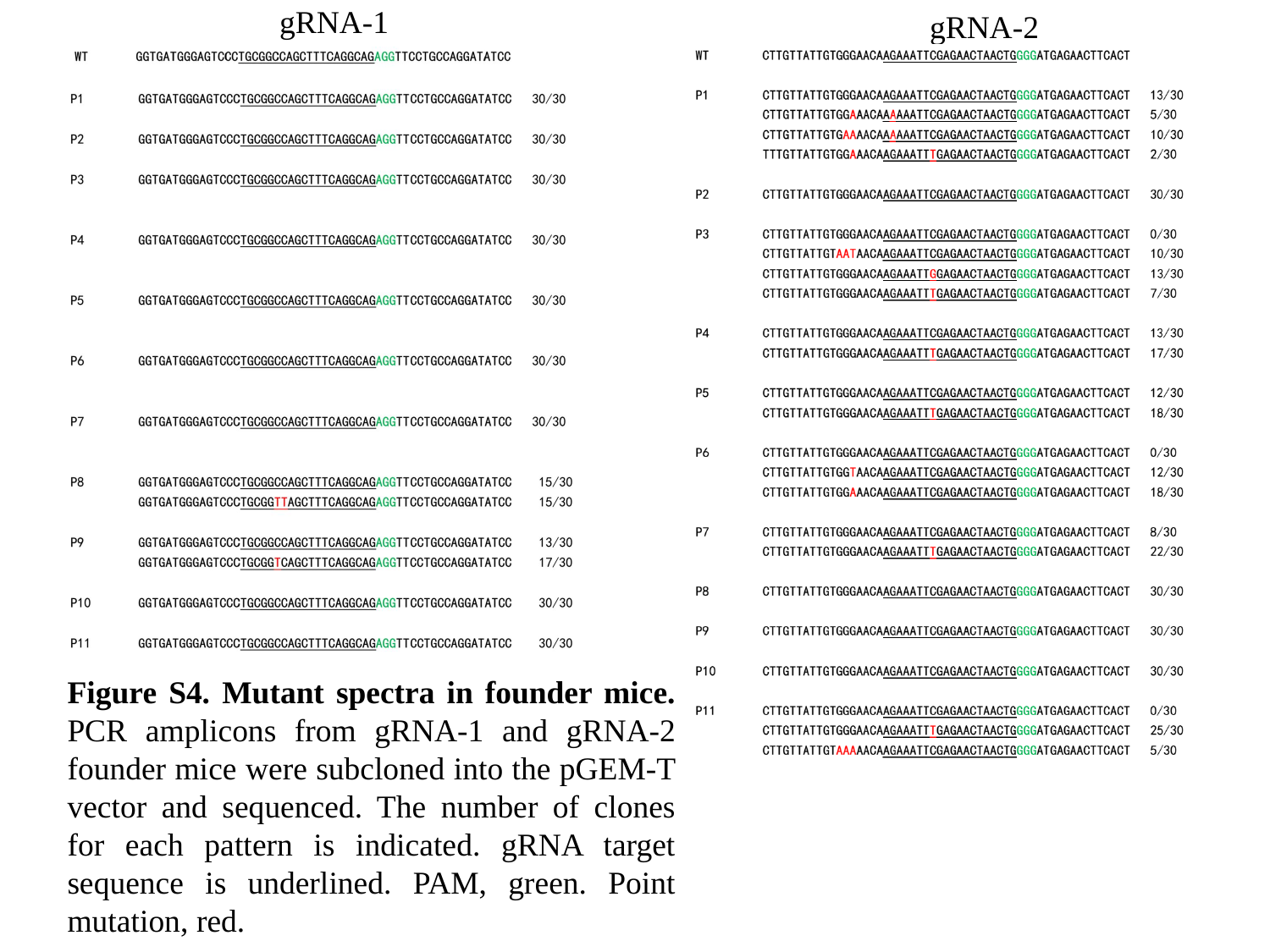

gRNA-1
gRNA-2
Figure S4. Mutant spectra in founder mice. PCR amplicons from gRNA-1 and gRNA-2 founder mice were subcloned into the pGEM-T vector and sequenced. The number of clones for each pattern is indicated. gRNA target sequence is underlined. PAM, green. Point mutation, red.

## Slide 5
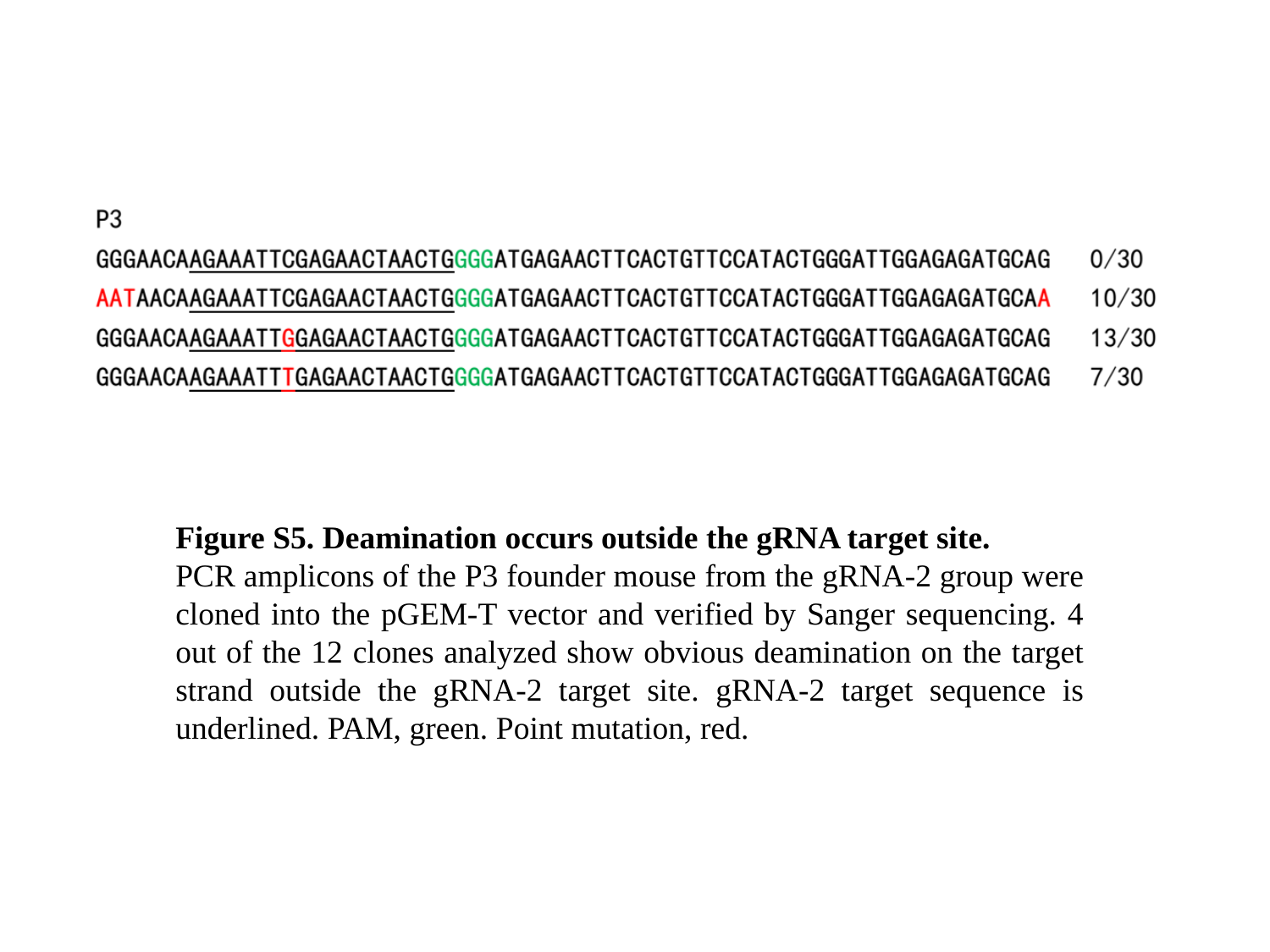

Figure S5. Deamination occurs outside the gRNA target site.
PCR amplicons of the P3 founder mouse from the gRNA-2 group were cloned into the pGEM-T vector and verified by Sanger sequencing. 4 out of the 12 clones analyzed show obvious deamination on the target strand outside the gRNA-2 target site. gRNA-2 target sequence is underlined. PAM, green. Point mutation, red.

## Slide 6
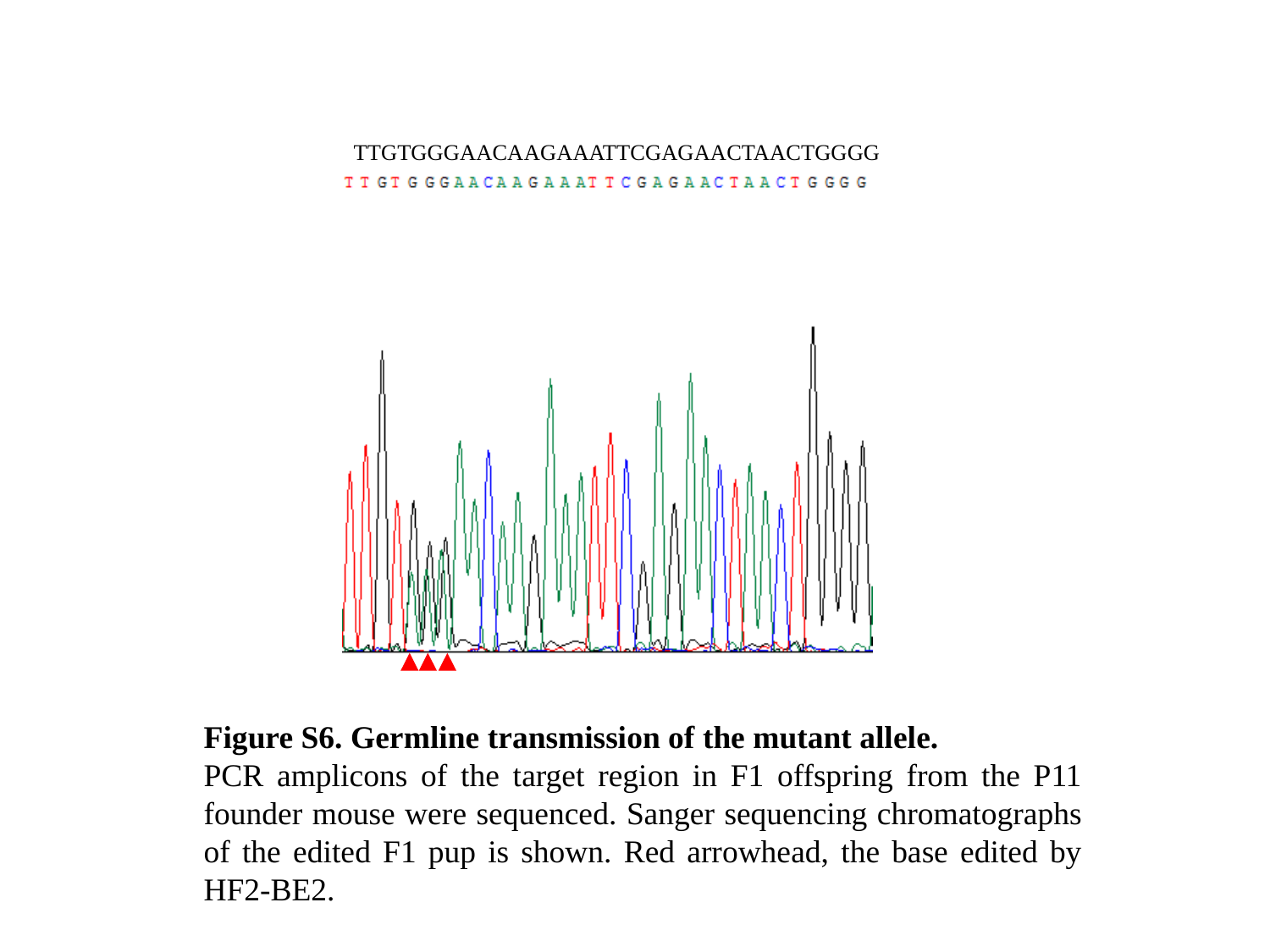

TTGTGGGAACAAGAAATTCGAGAACTAACTGGGG
Figure S6. Germline transmission of the mutant allele.
PCR amplicons of the target region in F1 offspring from the P11 founder mouse were sequenced. Sanger sequencing chromatographs of the edited F1 pup is shown. Red arrowhead, the base edited by HF2-BE2.
